# Computational Models of Neuron-Astrocyte Interactions Lead to Improved Efficacy in the Performance of Neural Networks

**DOI:** 10.1155/2012/476324

**Published:** 2012-05-09

**Authors:** Alberto Alvarellos-González, Alejandro Pazos, Ana B. Porto-Pazos

**Affiliations:** Departamento de Tecnologías de la Información y las Comunicaciones, Facultad de Informática, Universidade da Coruña, Campus de Elviña, 15071 A Coruña, Spain

## Abstract

The importance of astrocytes, one part of the glial system, for information processing in the brain has recently been demonstrated. Regarding information processing in multilayer connectionist systems, it has been shown that systems which include artificial neurons and astrocytes (Artificial Neuron-Glia Networks) have well-known advantages over identical systems including only artificial neurons. Since the actual impact of astrocytes in neural network function is unknown, we have investigated, using computational models, different astrocyte-neuron interactions for information processing; different neuron-glia algorithms have been implemented for training and validation of multilayer Artificial Neuron-Glia Networks oriented toward classification problem resolution. The results of the tests performed suggest that all the algorithms modelling astrocyte-induced synaptic potentiation improved artificial neural network performance, but their efficacy depended on the complexity of the problem.

## 1. Introduction

The behavior of the nervous system (NS) remains a mystery in many respects. The details of how the brain performs certain information processing tasks, such as classification, pattern recognition, and concept abstraction, are still unknown. Although it has long been thought that neurons were the only cells involved in complex cognitive processes, this thinking has changed. Recent discoveries show the importance of particular glial cells, called astrocytes, for information processing in the brain [1–8]. Abundant evidence suggests the existence of bidirectional communication between astrocytes and neurons and an important active role for the astrocytes in the physiology of the NS [1, 3–5]. This evidence has led to the proposal of a new concept in synaptic physiology, the tripartite synapse, which consists of three functional elements: the presynaptic and postsynaptic elements, and the surrounding astrocytes [2]. The communication between these three elements has highly complex characteristics, which seem to reflect more reliably the complexity of the information processing between elements of the NS. In order to understand the motives of this reciprocal signalling, we must know the differences and similarities that exist between their properties. Only a decade ago, it would have been absurd to suggest that these two cell types have very similar functions; now we realise that the similarities are striking from the perspective of chemical signalling. Both cell types receive chemical inputs that have an impact on their ionotropic and metabotropic receptors. Following this integration, both cell types send signals to their neighbours through the release of chemical transmitters. Both neuron-to-neuron signalling and neuron-to-astrocyte signalling show plastic properties that depend on the particular activity [1]. The main difference between astrocytes and neurons is that many neurons extend their axons over large distances and conduct action potentials of short duration at high speed, whereas astrocytes do not exhibit any electric excitability but conduct calcium spikes of long duration (tens of seconds) over short distances and at low speed. The fast signalling, and input/output functions in the central NS that require speed seem to belong to the neural domain. But what happens with slower events, such as induction of memories and other abstract processes, such as thought processes? Does signalling between astrocytes contribute to their control? As long as there are no answers to these questions, research must continue; the present work offers new ways to advance through the use of Artificial Intelligence (AI) techniques.

This work tries to add some new knowledge about the interaction of neurons and astrocytes regarding information processing, in both the brain and in computer AI systems. Hence, this is a multidisciplinary study. It tries to benefit both Neuroscience, by helping to understand the neuron-glia interaction, and AI, creating new computational methods for processing information. Including artificial elements that attempt to imitate astrocytes' behavior in Artificial Neural Networks (ANNs) has proven to present advantages in classification problems [9]. This inclusion gave rise to the so-called *Artificial Neuron-Glia Networks *(ANGNs) [9, 10]. In our previous work we have investigated the consequences of including artificial astrocytes, which mimic biologically defined properties involved in astrocyte-neuron communication, on artificial neural network performance. Using connectionist systems and evolutionary algorithms, we have compared the performance of ANN and ANGN in solving classification problems. We have shown that the degree of success of ANGN was superior to that of ANN. Analysis of the performance of ANN with different numbers of neurons or different architectures indicated that the effects of ANGN cannot be accounted for by an increased number of network elements but rather are specifically due to astrocytes. Furthermore, the relative efficacy of ANGN versus ANN increased as the complexity of the network increased [9]. It is important to note that our AI computational model does not account for the primary intrinsic physiological property of astrocytes, intercellular calcium waves in the astroglial network, such as in the work of Ikuta et al. [11, 12]. Instead of building an astroglial network with intercellular waves parallel to the neuronal network, and then analyzing their conjoint operation, we added single astrocytes to single neurons, allowing the astrocyte to increase the strength of the connections of the neuron with the next neuronal layer (see Section 2). At present, we are also modelling other types of astrocytic influence.

The neuron-astrocyte interaction in the ANGN implemented in our previous work constituted what we have called a neuron-glia algorithm. That first algorithm, which we have named *Attenuated effect of astrocyte* (see Section 3), tried to imitate a behavior observed between neurons and astrocytes in the hippocampus [6]. However, several mechanisms and physiological consequences of astrocyte-neuron communication occur in the brain. Under what conditions one specific modulatory effect takes place in a particular neural network remains unknown [8]. Therefore, in the present work we have researched whether other forms of interaction may help us understand what happens in the brain, and which may prove to be more or equally efficient in information processing in computers. We have modeled new and different neuron-astrocyte interactions, analyzing the results of Neuroscience experiments carried out in biological nervous systems [4–8]. This has led to the implementation of different neuron-glia algorithms for training and validation of feed-forward multilayer ANGN used in resolution of classification problems. For simplicity, our work focused on modelling astrocyte-induced synaptic potentiation, as in our previous study [9]. The results obtained using these new algorithms allowed comparisons between them and the observation of neuron-glia interactions that, so far, obtain the best results for each problem.

This paper is organised into the following sections. Section 2 introduces the ANGN and explains its overall behavior, and its differences with multilayer networks without artificial astrocytes. Section 3 details the implementation of neuron-glia algorithms created for the study of neuron-astrocyte interaction.  Section 4 explains the simulations performed applying the created algorithms to two problems, *simulation of a multiplexor device* and *iris flower classification*, and shows the results obtained from these simulations. Finally, Section 5 summarizes the discussion and conclusions of this study and explains the work that is being developed as a continuation of this research.

## 2. Artificial Neuron-Glia Networks

**Figure 1:**
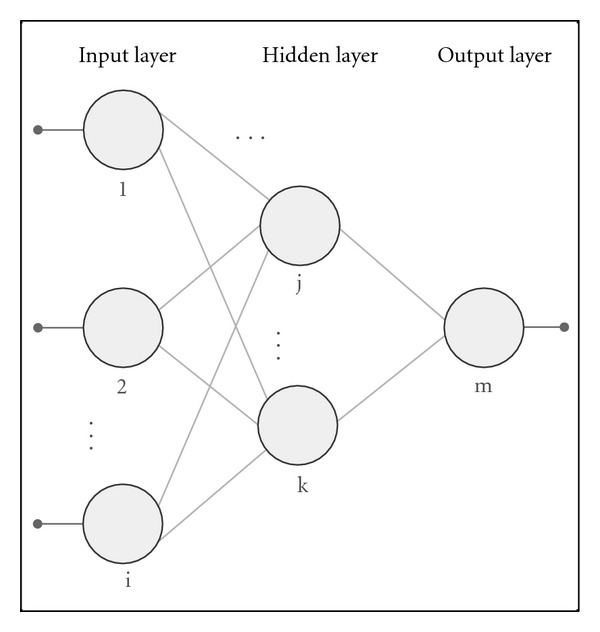
Artificial Neural Network structure.

**Figure 2:**
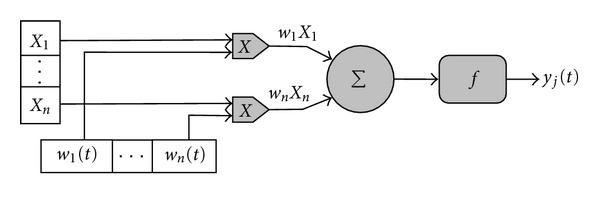
Structure of an artificial neuron.

ANNs are interconnected neuron models that simulate the behavior of biological neural networks [13, 14] (see Figure 1). The neuron is the basic information-processing unit in these networks (see Figure 2).

The connectionist branch of AI carries out the study of ANN. Researchers in this area have designed and built different types of ANN; these systems are different in their topology, dynamics, and behavior of their constituent elements separately and together (the system as a whole). Many advances have been made in those aspects, but there are many limitations in the areas of processing speed and computational complexity of the connectionist systems.

The connection between two neurons is a directional one. Hence, one neuron *a* is the source of the connection and the other  *b* is the destination neuron. A value is associated with the connection. This value is known as *weight* and it determines the influence of the connection in the activation of the destination neuron.

A neuron *j*, is characterized by *n* inputs, with signals *x*_1_ to *x*_*n*_ and weights *w*_1_ to *w*_*n*_ associated with the inputs.

The signals may come from other neurons or may be the input signals of the network. The output of the neuron *j* is given by the application of the transfer function *f*∶ℝ → ℝ [15] to the sum of the inputs adjusted by its associated weight:

(1) $$y_{j}\left(t\right)=f\left(\overset{n}{\underset{i=1}{\sum }}w_{i}x_{i}\right).$$

**Figure 3:**
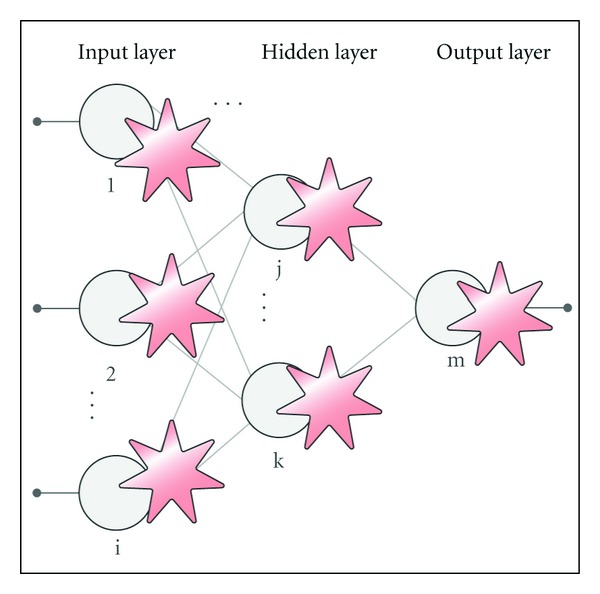
Artificial Neuron-glia network structure.

An ANGN extends the ANN architecture by including a new kind of processing element, the artificial astrocyte [9, 10, 16–18] (see Figure 3).

**Figure 4:**
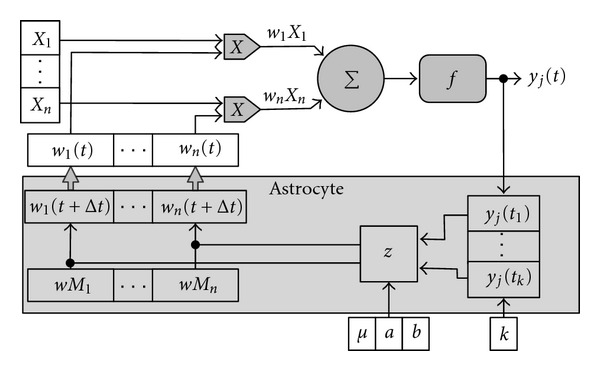
Astrocyte representation.

An artificial astrocyte is associated with a neuron and controls its activity (see Figure 4). The astrocyte modifies the weight of the connections it is associated with (input connections, output connections, or both), depending on the activity of the neuron. A range of values is also associated with a weight, known as a weight limit (wM). The astrocyte controls the activity of the *j* neuron by using a counter. That counter records the times that the neuron fires.

Due to the lack of knowledge regarding the specific characteristics of the modifications that astrocytes make in neuronal connections, we implemented different *neuron-glia algorithms* (see Section 3) to simulate the behavior which astrocytes of the brain are presumed to have, considering the observations made on the nervous systems of living organisms [1–4]. Glutamate released in the extracellular space by an astrocyte or a presynaptic neuron can affect another astrocyte, another presynaptic neuron, or a postsynaptic neuron. If the glutamate that reaches a postsynaptic neuron proceeds directly from a presynaptic neuron, the action potential (AP) takes place more rapidly and ends more or less quickly. If there has also been a release of glutamate by an astrocyte that was activated by the glutamate of a presynaptic neuron, more AP will take place [1]. The activation of astrocytes is a slow process, if we compare it with neural activity [4]. The same conclusion can be drawn from their effect on the synapse between two neurons, whose neurotransmitters activated the astrocyte, and which is one thousand times slower than the propagation of the impulse by the neurons. This slowness has led to the presentation to the ANGN of each training pattern during more than one cycle or iteration. If it imitates this slowness, the ANGN will need *k* cycles or iterations to process each input pattern (see Figure 4).

We must also consider that the contribution of the astrocytes to the weights of the ANGN connections takes place according to a time factor, given the fact that they act slowly and their responses are non-linear. It would be interesting to know how astrocytes affect the connectionist system, considering their influence on the synapses according to the activities of the neurons over the course of time. The more intense the activity of the neurons, the larger the influence of the astrocyte on a connection.

The behavior of an astrocyte is determinate by the parameters *k* ∈ *ℕ*∖{0}, *μ* ∈ [1, *k*] and *a*, *b* ∈ [0, 1]. Each instance or input pattern that is used for training, validating or testing the artificial net is processed *k* times (iterations). The astrocyte registers the activity of the neuron during the *k* iterations, applying a function *u*∶ℝ → *ℤ* over the output of the neuron *y*_*j*_(*t*), where *u* indicates if the neuron has fired *u*(*x*) = 1 or not *u*(*x*) = −1:

(2) $$u\left(x\right)=\{\begin{array}{cc} -1, & x\leq 0,\\ 1, & x>0. \end{array}$$

Hence the astrocyte has a register of the neuron's activity with a temporal window of *k* instants of time (an iteration lasts one instant of time). Observing this activity, the astrocyte will modify the weight of its associated neuronal connections when the counter of the activity of the neurons reaches the value *μ*. Figure 4 shows how the input neuronal connections are modified. An astrocyte may also modify output neuronal connections or both

(3) $$w_{i}\left(t+\Delta t\right)=w_{i}\left(t\right)+\Delta w_{i}\left(t\right),$$

where

(4) $$\Delta w_{i}\left(t\right)=\left|w_{i}\left(t\right)\right|\;z\left(t\right),$$

and *z*∶*ℕ*∖{0} → ℝ is a function defined as

(5) $$z\left(t\right)=\{\begin{array}{cc} a, & r_{j}\left(t\right)=\mu ,\\ -b, & r_{j}\left(t\right)=-\mu , \end{array}$$

with *r*_*j*_∶*ℕ*∖{0} → [−*μ*, *μ*]  being the function that returns the number of times a neuron has fired. If the neuron was active *μ* times, the weights of the connections will be increased by a percentage *a*, while they will be decreased by a percentage *b* if the neuron remained inactive during *μ* iterations.

## 3. Neuron-Glia Algorithms

The six algorithms implemented were different in two aspects: the specific implementation they make of the *r*_*j*_ function, and whether or not they respect the weight limit when the neuronal connection is being modified. The different implementations of the *r*_*j*_ function of each algorithm are explained in its corresponding subsection. Different approaches regarding the modification of the connections weight are also explained.

### 3.1. Consecutive Activations, Weight Limited

The astrocyte respects the weight limit of the connections:

(6) $$w_{i}\left(t+∆t\right)=\min\left\{w_{i}\left(t\right)+∆w_{ji}\left(t\right),{wM}_{i}\right\}.$$

This algorithm contemplates only consecutive neuron activations; if the neuron reaches the activity or inactivity level that makes the astrocyte act, the activity counter is restarted. The neuronal activity level, following these restrictions, is given by the following function:

(7) $${\text{r}}_{\text{j}}\left(\text{t}\right)=\{\begin{array}{cc} u\left(y_{j}\left(t\right)\right)+r_{j}\left(t-1\right), & t>0,\;u\left(y_{j}\left(t\right)\right)=u\left(y_{j}\left(t-1\right)\right),\\ & r_{j}\left(t-1\right)\in \left(-\mu ,\mu \right),\\ u\left(y_{j}\left(t\right)\right), & \text{in}\;\text{other}\;\text{case}. \end{array}$$

### 3.2. Consecutive Activations, Weight Unlimited

The behavior of this algorithm is the same as the previous one, except that in this case the astrocyte will not respect the limit weight of the connections; hence they can reach any value:

(8) $$w_{i}\left(t+\Delta t\right)=w_{i}\left(t\right)+\Delta w_{i}\left(t\right).$$

### 3.3. Nonconsecutive Activations, Weight Limited

The astrocyte respects the weight limit of the connections

(9) $$w_{i}\left(t+\Delta t\right)=\min\left\{w_{i}\left(t\right)+\Delta w_{ji}\left(t\right),{wM}_{i}\right\}.$$

In this algorithm the neuron activations need not be consecutive. If the neuron reaches the activity or inactivity level that makes the astrocyte act, the activity counter is restarted. The neuron activity level, following these restrictions, is given by the following function:

(10) $$r_{j}\left(t\right)=\{\begin{array}{cc} u\left(y_{j}\left(t\right)\right)+r_{j}\left(t-1\right), & t>0,\;r_{j}\left(t-1\right)\in \left(-\mu ,\mu \right),\\ u\left(y_{j}\left(t\right)\right),\; & \text{in}\;\text{other}\;\text{case}, \end{array}$$

Having the activity of the neuron not required to be consecutive gives rise to this result: if an astrocyte increments the weight of a connection of a neuron, it indicates that the neuron fired *μ* iterations more than it remained inactive. If an astrocyte decrements the weight of a connection to a neuron, it indicates that the neuron fired *μ* iterations less than it remained inactive.

### 3.4. Nonconsecutive Activations, Weight Unlimited

The behavior of this algorithm is the same as the previous one, except that in this case the astrocyte will not respect the limit weight of the connections; hence they can reach any value:

(11) $$w_{i}\left(t+\Delta t\right)=w_{i}\left(t\right)+\Delta w_{i}\left(t\right).$$

### 3.5. Attenuated Effect of Astrocyte

In this algorithm, the astrocyte will not respect the limit weight of the connections:

(12) $$w_{i}\left(t+\Delta t\right)=w_{i}\left(t\right)+\Delta w_{i}\left(t\right),$$

and the activity of the neuron need not be consecutive

(13) $$r_{j}\left(t\right)=\{\begin{array}{cc} u\left(y_{j}\left(t\right)\right)+r_{j}\left(t-1\right), & t>0,\;r_{j}\left(t-1\right)\in \left(-\mu ,\mu \right),\\ r_{j}\left(t-1\right), & t>0,\;r_{j}\left(t-1\right)\in \left\{-\mu ,\mu \right\},\\ u\left(y_{j}\left(t\right)\right), & \text{in}\;\text{other}\;\text{case}. \end{array}$$

The major difference with the previous algorithms stems from the management of the activity counter of the neuron: when the neuron reaches the activity level {−*μ*, *μ*} that makes the astrocyte modify its neuronal connections, the activity counter is not set to zero (it retains the value). This behavior has a noticeable consequence in the modification of the connections weight: when the point at which an astrocyte modifies the weight of the connections is reached in a given iteration and the neuron fires again in the next iteration, the astrocyte will increase the connections weight of the neuron again. The behavior when the neuron remains inactive is similar, with the outcome being the weight is decreased.

In the previous algorithms, having the activation counter be set to zero, the counter needed to reach the value {−*μ*, *μ*} again for the astrocyte to act (thus a minimum of *μ* iterations of neuronal activity/inactivity are required). This behavior implies an extra reinforcement on those neurons that fire the most, it also makes the astrocytic effect last longer, and disappear only gradually over time.

### 3.6. Global Processing Effect

**Figure 5:**
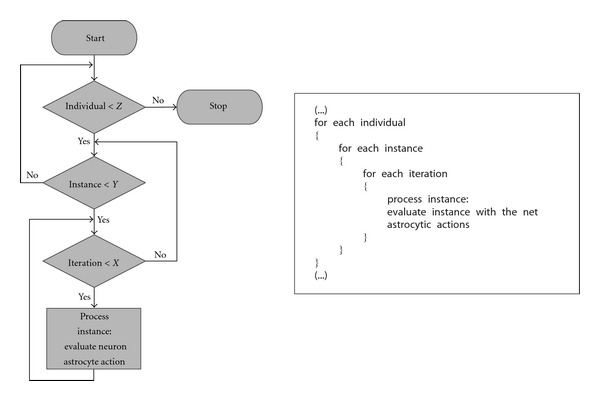
Flow chart and pseudocode of the five Neuron-Glia algorithms.

In the previous algorithms, each instance was processed a certain number of iterations to try to simulate the delay in glial system functioning (an order of magnitude of seconds) with respect to neuronal functioning (an order of magnitude of milliseconds) (see Figure 5) [19, 20].

**Figure 6:**
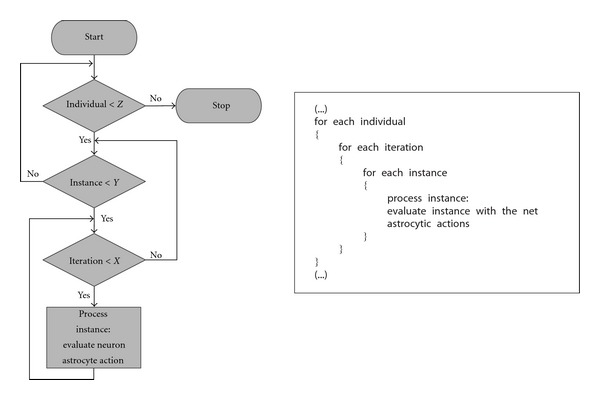
Global processing effect flowchart and pseudocode.

This algorithm named *Global processing effect* was created under the assumption that the way the brain works is different than the way it was being simulated. Instead of processing each instance during k iterations, this algorithm considers the instances as a whole, and the net processes the whole set of instances during *k* iterations (see Figure 6). For example, this algorithm considers the following: when visual information (i.e., a scene) is being processed by the brain, it obtains a sequence of images and this sequence is processed as a whole. Common characteristics are extracted from these images to give the scene meaning. The previous algorithms would consider each image independently and would try to extract characteristics from each one separately.

The way this new algorithm modifies the weights of the connections is different from the previous algorithms. with where *n* is the number of instances (for training/validating/testing), this algorithm evaluates the *n* instances during *k* iterations. The weights are modified using

(14) $$w_{i}\left(t+\Delta t\right)=w_{i}\left(t\right)\;\Delta w_{i}\left(t\right),$$

where

(15) $$\Delta w_{i}\left(t\right)=\frac{n+\mathrm{sgn}\left(w_{ji}\left(t\right)\right)s}{n},$$

With *s* being the number of times the neuron was active during *k* iterations. With this behavior, the modification of connections weight of the neuron is directly proportional to the neuron's activity.

## 4. Results

To evaluate the functioning of the neuron-glia algorithms, ANGN using our different algorithms was compared with ANN (without artificial astrocytes) trained only by using Genetic Algorithms (GAs). The ANGN training method is a hybrid one. It is composed of two learning phases: an unsupervised learning phase (where a selected neuron-glia algorithm is used) and a supervised learning phase (where GAs are applied using the MSE calculated in the unsupervised phase) [9, 10, 16]. The networks to be compared (seven networks: ANGN with 6 different unsupervised algorithms and the ANN—see Table 1) were trained to solve two classification problems of increasing complexity. The problems were taken from the UCI Machine Learning Repository [21]: the *simulation of a multiplexed device* problem and the *Iris Flower *problem.

The network architecture chosen for each problem was selected based on good results achieved in previous work by Rabuñal et al. [22, 23]. Anyway, what matters is not to have the best architecture. The important issue is to have the same architecture in all the networks to be compared, in order to test effects caused only by the inclusion of artificial astrocytes. Anyway, we had shown in our previous work [9] that by increasing the number of neurons and layers, the effect of artificial astrocytes becomes even more beneficial.

Regarding the parameters of neuron-glia algorithms in ANGN, four different combinations of iterations *k* (4, 6, or 8) and activations *μ* (2 or 3) were considered for each algorithm, in particular (4-2, 6-2, 6-3 y 8-3); after doing preliminary tests [16], the combinations that obtained the best results were selected. The same parameters (*k*, *μ*, *a*, and *b*) were used for all the artificial astrocytes in each simulation. Each simulation also used a 25% weight increment (a) and a 50% weight decrement (b). This decision was based on the good results obtained in previous work [16]. Biological knowledge supports this choice, because a lower increment than a decrement, when there is no constant activity, reinforces the connections of those neurons that show a constant activity [24].

Regarding the GA parameters, they were also chosen in contemplation of previous work from Rabuñal et al. [22, 23]. Those parameters were not intended to obtain the best results in every problem but to use the same parameters in the connectionist systems to be compared. A population of 100 individuals was used in the GA. The individual selection algorithm chosen was “Montecarlo” and the substitution method was the Darwinian substitution. Regarding individual breeding, a single crossover point was used. The crossover rate was set to 90% and the mutation rate to 10%.

The simulations were performed by means of a tool we have implemented with Borland DELPHI and Visual C++ languages. The tests were run in an AMD Athlon PC, with 1 GB of RAM and Windows XP OS.

### 4.1. Simulation of a Multiplexor Device (MUX)

**Figure 7:**
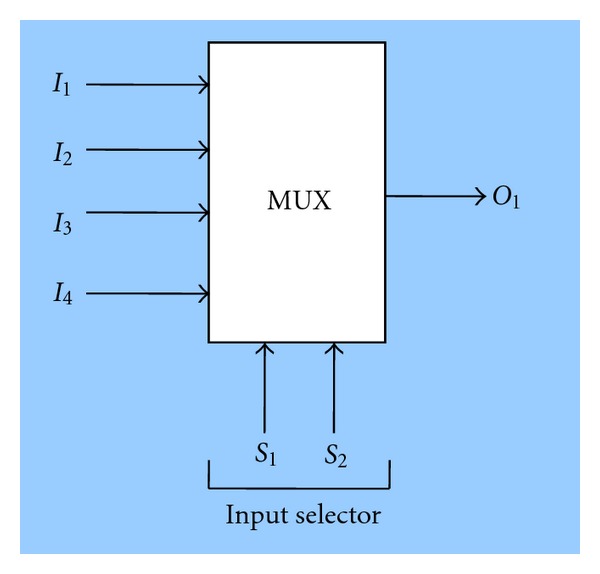
MUX device.

We decided to carry out the first tests over a well-known multilayer architecture, which solves a simple classification problem: the simulation of an electronic device called MUltipleXor (MUX) with four inputs and one output (see Figure 7). For the resolution of this problem a three-layer network was used: six neurons in the input layer, four in the hidden layer and one neuron in the output layer. The activation function used for all the neurons in the network was a threshold function with a threshold value *θ* = 0.5. The output values of this problem are boolean. The limit weight for all the neurons was set to one. The 64 instances available for this problem were divided into two sets: 58 instances for training, and six instances where different classes were selected for the purpose of checking the generalization capacity. All simulations were executed over 4000 generations.

All implemented neuron-glia algorithms were tested with the MUX problem (see Table 1).

Each algorithm was executed using ten different populations of connection weights. For each population and algorithm the best values (those with higher validation accuracy) were chosen from among the aforementioned four *μ* and *k* combinations used.  Table 2 shows the mean values for each algorithm. These results show that in all cases but two, ANGN achieved a lower training error, and in all cases a higher validation accuracy was achieved by ANGN with respect to ANN results.

The number of times each algorithm achieved the best results for each measurement was analyzed (fewer generations, lower training error, higher validation accuracy, or less time).  Table 3 shows that ANGN achieved the best results more frequently, considering the validation accuracy, despite taking more time to achieve it.

### 4.2. Iris Flowers Classification (Iris)

We wanted to prove our algorithms with a problem related to a much more complex domain than MUX, a problem to test the algorithms where the ANGN is dealing with multiple classification tasks. In contrast to the MUX problem, the IRIS flower problem uses continuous input values, different activation functions in artificial neurons of different layers, and twice as many training patterns. It consists in identifying a plant's species: Iris setosa, Iris versicolor, or Iris virginica. This case has 150 examples with four continuous inputs which stand for four features about the flower's shape. The four input values represent measurements in millimetres of petal width, petal length, sepal width, and sepal length. The learning patterns have been found to have four inputs and three outputs. The three outputs are Boolean ones, representing each Iris species. By doing it in this manner (three boolean outputs instead of a multiple one), additional information can be provided about whether the system's outputs are reliable or not. That is, due to the outputs' intrinsic features, only one of them must possess the true value, standing for the type of flower it has classified, while the rest have a false value. Therefore, if two or more outputs have true values, or if all of them are false, we may conclude that the value classified by the system is an error and the system cannot classify that case. The values corresponding to the four input variables have been normalized in the interval (0–1) so that they are dealt with by the ANGN.

For the resolution of this problem a three-layer network was used: four neurons in the input layer, five in the hidden layer, and three neurons in the output layer (one neuron per iris class). The activation function used for all the neurons in the network was a hyperbolic tangent, except for the output layer neurons, where a threshold function with a threshold value *θ* = 0.5 was used. The limit weight for all the neurons was set to 1. The 150 instances available for this problem were divided into two sets: 2/3 for training and 1/3 for validation; the training set is composed of 1/3 of instances of each class. All simulations were executed over 2000 generations.

Although, as mentioned previously, what matters is having the same architecture, we have established this architecture and these parameters, which were obtained by our research group in previous work [22, 23]. By using ANN with these features, and training exclusively by means of GA, Rabuñal et al. [22, 23] reached an adjustment better than the previous best example of work for solving the IRIS flower with ANN, in which Martìnez and Goddard [25] used BP for the training and a hidden neuron more than Rabuñal et al. These good results demonstrated the efficacy of GA for simplifying and solving this problem. We compared our new ANGN with ANN trained exclusively by means of GA.

The algorithm named *Attenuated Effect of Astrocyte* was chosen because it achieved the best results in the MUX problem (good mean validation accuracy, and the best results achieved more often). With the aim of adding some variability to the results, two configurations of this algorithm were used: one with *k* ∈ [4,6, 8] and *μ* ∈ [2,3], and another with *k* ∈ [3,5, 7] and *μ* ∈ [2,3]. The latter is called *Attenuated Effect of Astrocyte 2 in* Table 4.

Another algorithm was chosen from the remaining ones to test its behavior against a more complex problem. The less restrictive algorithm was chosen (in terms of both activations of neurons and restrictions on modifications of weights of connections): “Not consecutive activations, weight unlimited”.

As with the MUX problem, each algorithm was executed using ten different populations of weights. For each population and algorithm the best values (those with higher validation accuracy) were chosen among the four *μ* and *k* combinations used.  Table 5 shows the mean values for each algorithm. These results show that in all cases but two, ANGN achieved a lower training error, and in all cases a higher validation accuracy was achieved by ANGN with respect to results with ANN.

Regarding the number of times each algorithm obtained the best results, Table 6 shows that ANGN achieved the best results more times, considering the validation accuracy, despite taking more time to achieve it.

## 5. Discussion and Conclusions

All the implemented algorithms have tried to emulate, in the ANGN, the potentiation of synaptic connections that take place in the brain caused by astrocytes, due to high synaptic activity. The first five algorithms emulate astrocytic behavior in a similar way, just changing the restrictions on the weights changes and the consecutive or not consecutive nature of synaptic activity. Unlike in the first five algorithms, the sixth algorithm operates in a fairly different manner: it considers global information processing rather than an individual instance.

ANGN implemented using these six algorithms (thus including artificial astrocytes that simulate the potentiation of the connections and penalize the lack of activity) improved the ANN that did not include artificial astrocytes. It is worth noticing the difference in efficacy between the first five algorithms and the sixth, which achieved the worst results.

It was also observed that the *Not consecutive, weight unlimited* algorithm did not achieve the best results in the simpler problem (MUX) but did achieve the best results in the more complex problem (Iris) with a more complex net architecture. This suggests that the net (and problem) complexity influences the behavior of an algorithm, rendering an algorithm more appropriate for one problem or another depending on the problem's complexity. This allows the conclusion that a specific behavior of astrocytes is better suited to some problems rather than to others, depending on the problem's complexity and characteristics. This behavior agrees with the biological behavior of astrocytes in the brain. Sometimes they have more influence than at others. Moreover, it has been observed that the number of astrocytes is higher in more complex brain areas, and they have more influence in them. The highest ratio of glia-to-neurons is found at the top of the phylogenetic tree, in the human brain; this leaves us with the question as to whether astrocytes are key regulatory elements of higher cortical functions [3].

In any case, to prove the hypothesis obtained, more tests are being performed with the Iris problem and with other problems (starting from the preliminary test and the algorithms developed in this work). Therefore, the development of models of astrocyte-neuron interaction that incorporate the richness of biological interactions, for example, astrocyte-induced synaptic depression, or depression and potentiation altogether, is being undertaken to test whether they provide similar, or even better, results in neural network performances.

This work attempts to assist both AI and Neuroscience. the former by creating a new kind of information processing element for neural networks, the latter by contributing ideas that hope to, somehow, guide investigations in Neuroscience toward understanding the behavior of astrocytes in the nervous systems of living organisms. All the algorithms presented could attempt to be translated in a laboratory for Neuroscience. They may mimic physiological dynamics that occur in the brain.

Understanding the importance of astrocytes in brain function, and learning how communication occurs between neuronal networks and astrocyte networks at a microscopic level, is crucial for the understanding of the synergic behavior of cerebral regions. It is known that astrocytes are not present in the same proportion in all brain regions, which may influence the existence of different modes of interaction between one and other areas. The behavior of astrocytes in some regions is just beginning to be analyzed, in which its effects on information processing have never been studied, such as cerebral cortex. Computational models to study brain circuitry connectivity at a microscopic level will allow an understanding of what happens at a macroscopic level. Moreover, given the efficacy of ANGN in processing information, they could provide a double benefit to this area of study. Since they can be used to classify and recognize patterns, ANGN will be tested in the near future as a data analysis tool for helping to detect any characteristic or neurological disorder in brain signals acquired in different modalities, such as electroencephalography, and magnetoencephalography.

## Figures and Tables

**Table 1:** Methods name summary.

Method	Name
1	Consecutive, weight limited
2	Consecutive, weight unlimited
3	Not consecutive, weight limited
4	Not consecutive, weight unlimited
5	Attenuated effect of astrocyte
6	Global processing effect
7	ANN

**Table 2:** MUX mean results.

Method	Generation	Training error (ECM)	Training standard deviation (%)	Validation accuracy (%)	Validation standard deviation	Time
1	222	0,132	0,056	86,25	8,75	0:00:21
2	282,1	0,091	0,027	81,25	10,08	0:00:38
3	345,2	0,125	0,059	86,25	8,75	0:00:29
4	355,6	0,083	0,021	81,25	10,08	0:00:45
5	681,3	0,108	0,060	86,25	10,38	0:01:08
6	563,6	0,096	0,057	76,25	3,75	0:00:33
7	521,8	0,101	0,051	62,5	9,68	0:00:07

**Table 3:** MUX results summary.

Method	Generation	Training error	Validation accuracy	Time	Total
1	3	1	3	1	8
2	2	3	3	1	9
3	3	1	3	1	8
4	2	3	3	1	9
5	1	3	4	0	8
6	0	4	0	3	7
7	3	3	0	6	12

**Table 4:** Iris methods summary.

Method	Name
1	Not consecutive, weight unlimited
2	Attenuated Effect of Astrocyte
3	Attenuated Effect of Astrocyte 2
4	ANN

**Table 5:** Iris mean results.

Method	Generation	Training error (ECM)	Training standard deviation	Validation accuracy (%)	Validation standard deviation	Time
1	693,9	0,065	0,023	78,2	5,76	0:03:17
2	486,8	0,151	0,079	72,4	5,64	0:02:44
3	868,6	0,155	0,098	70,2	8,17	0:04:30
4	166,6	0,371	0,051	56	4,29	0:00:09

**Table 6:** Iris results summary.

Method	Generation	Training error	Validation accuracy	Time	Total
1	0	8	7	0	15
2	0	0	2	0	2
3	1	2	2	1	6
4	9	0	0	9	18
